# Providing diabetes education to patients with chronic kidney disease: A survey of diabetes educators in Ontario, Canada

**DOI:** 10.1177/26335565211062758

**Published:** 2021-12-13

**Authors:** Kristin K Clemens, Alexandra M Ouédraogo, Selina L Liu, Paulina Bleah, Amanda Mikalachki, Tamara Spaic

**Affiliations:** 1 Department of Medicine, Division of Endocrinology and Metabolism, 6221 Western University, London, ON, Canada; 2 Centre for Diabetes, Endocrinology and Metabolism, St. Joseph’s Health Care London, London, ON, Canada; 3 Lawson Health Research Institute, London, ON, Canada; 4 50010 ICES Western, London, ON, Canada; 5 University Health Network, Division of Nephrology, Toronto General Hospital, Toronto, ON, Canada; 6 Primary Care Diabetes Support Program, London, ON, Canada

**Keywords:** Diabetes educator, chronic kidney disease, survey, challenges, diet, self-management, education

## Abstract

**Background:**

Patients with diabetes and chronic kidney disease (CKD) have complex diabetes care needs. Diabetes educators can play an important role in their clinical care.

**Aim:**

To understand diabetes educators’ experience providing diabetes support to patients with CKD and elicit their view on the additional care needs of this population.

**Methods:**

We conducted a quantitative online survey of diabetes educators between May 2019 and May 2020. We surveyed English-speaking educators actively practicing in Ontario, Canada for at least 1 year. We recruited them through provincial Diabetes Education Programs and Diabetes Education Section Chairs of Diabetes Canada.

**Results:**

We made email contact with 219/233 (94%) Diabetes Education Programs and 11/12 (92%) provincial Diabetes Canada Section Chairs. 122 unique diabetes educators submitted complete surveys (survey participation rate ∼79%). Most worked in community education programs (91%). Almost half were registered nurses (48%), and 39% had practiced for more than 15 years. Respondents noted difficulty helping patients balance complex medical conditions (19%), faced socioeconomic barriers (17%), and struggled to provide dietary advice (16%). One-third were uncertain of how to support those receiving dialysis. Eighty-five percent felt they needed more training and education to care for this high-risk group. When asked about the care needs of patients with CKD, almost all (90%) felt that patients needed more diabetes support in general. Improvement in care coordination was most commonly suggested (38%).

**Conclusions:**

In this study of the diabetes educators’ experience treating patients with diabetes and CKD, respondents noted numerous challenges. There may be opportunities to better support both diabetes care professionals, and patients who live with multiple medical comorbidities.

## Introduction

Diabetes educators provide self-management support and education to patients across the spectrum of diabetes: type 1 and 2 disease, younger and older adults, and those who live with other medical comorbidities.^1^ Educators aim to provide patients with the skill and knowledge to live with diabetes, with a goal of reducing the risk and progression of diabetes-related complications.^1^

While self-management support and education about diabetes treatments, prevention of complications, and the importance of diet and exercise can be helpful to patients,^2,3^ there can be challenges, particularly when caring for those with comorbid diabetes and chronic kidney disease (CKD, typically defined by an estimated glomerular filtration rate (eGFR) <60 mL/min/1.73 m^2^).^4^ Patients with CKD and diabetes are at 10-fold higher risk of hypoglycemia than those with diabetes without CKD.^5^ They have altered drug metabolism which limits use of many oral and injectable diabetes medications, and face numerous dietary restrictions (i.e., limitations on potassium- and phosphate-containing food) which can make dietary counseling difficult.^1,6^ Moreover, patients with CKD frequently have lower income levels, lower levels of education, and more deprivation and dependency.^3,7,8^ These socioeconomic barriers not only limit patients’ access to medications and technology but contribute to lower levels of health literacy, difficulty with disease understanding, and suboptimal self-management and decision-making skills.^9^ People with diabetes and CKD, particularly those with advanced disease or receiving dialysis, also experience gaps in their diabetes care. They are under-screened for diabetes-related complications including retinopathy, and have suboptimal hemoglobin A1c and lipid testing.^7,10^ While care gaps might be due to patient (e.g., not being aware of the importance of screening), and health systems factors (systems are poorly organized for patients with multimorbidity),^11^ care providers might also have difficulty supporting these individuals due to an overall lack of expertise in multimorbidity, or limited evidence-based guidelines for the treatment of multimorbid populations.^12–14^

To close healthcare gaps and improve outcomes in those with diabetes and CKD, it is important to not only understand patient care needs and expectations^8^ but the views of key stakeholders involved in their healthcare. While there have been qualitative and quantitative studies to examine the care perspectives of family doctors and specialists who manage diabetes and CKD,^12,15^ there is no literature on the perspective of allied healthcare providers including diabetes educators, personnel who play an important role in their interdisciplinary care team.^16^ Moreover, while there have been efforts to develop and study special programs/services to support patients with diabetes and CKD (e.g., outreach support in primary care, targeted self-management and education, and interdisciplinary care clinics),^17^ the perspective of allied health care providers on the clinical needs of this population is unclear.

In this study, we aimed to understand (1) diabetes educators’ experience treating patients with CKD and (2) the educators’ views on the diabetes-related needs of this population. In an effort to develop patient-centered feasible, and innovative programming to better support these patients,^18^ as secondary aims we asked educators about (1) the availability of specialized diabetes support programs for patients with CKD in their communities, and (2) educators’ interest in participating in new care models for this patient group.

## Methods

### Design, setting and participants

We conducted a quantitative online survey of English-speaking diabetes educators actively practicing in Ontario, Canada for at least 1 year. Educators who could not complete the survey by study closure (May 2020) were excluded.

### Sampling and recruitment

We used convenience sampling to recruit diabetes educators starting in May 2019. We recruited participants in two ways; through provincial Diabetes Education Programs (DEPs) and Diabetes Canada Chapter Chairs.

In Ontario, DEPs are outpatient facilities where diabetes nurse educators and registered dieticians provide diabetes education and self-management support to those aged 18 years and older.^19^ DEPs are distributed across the province of Ontario.

As a first recruitment method, we sent an introductory email to all Ontario DEPs with publicly available email addresses available on thehealthline.ca or 211ontario.ca (search engines for health and community services in Ontario). If our initial recruitment email failed to transmit to the DEP, we telephoned them to verify email addresses using publicly available telephone numbers.

In our introductory email, we summarized our study, provided our rationale and objectives, and asked DEPs to either (1) share the email addresses of educators who worked in their center (if educators had previously agreed to be contacted by researchers) or (2) send our introductory email with survey link directly to educators on behalf of our research team.

As a second recruitment method, we emailed Ontario Chairs of all Diabetes Educator Sections of Diabetes Canada (Canada’s national diabetes organization, www.diabetes.ca). Chairs are responsible for sharing Diabetes Canada updates and strategic priorities with Section members, and informing them of certified medical education opportunities including conferences. At the time of the study, there were 12 Ontario Chairs. We used Diabetes Canada’s email network (Timed Right) to send Chairs our introductory email and survey link. We asked them to share our survey with Section members by email or at a scheduled Chapter meeting.

To facilitate survey completion, we sent a reminder email to DEPs, diabetes educators (where individual emails were provided by DEPs) and Section Chairs approximately 1 month after initial contact. Our recruitment processes are summarized in Figure 1 of the Supplemental Material.

**Figure 1.**
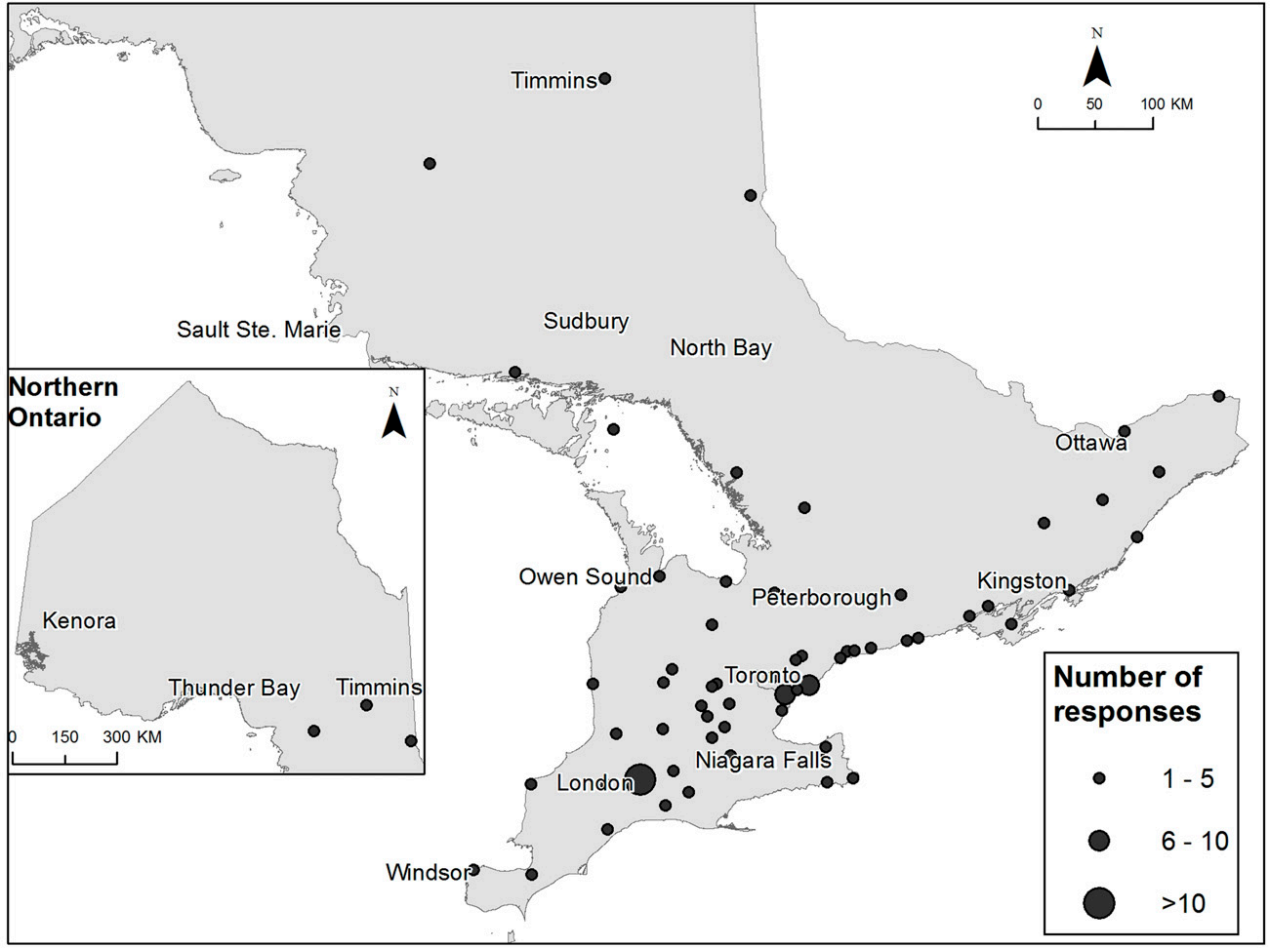
Location of practice of diabetes educator participants across Ontario, Canada.

### Sample size

We estimated that surveying 90 educators of the ∼2900 certified to practice in Ontario,^20^ would provide proportion estimates with 95% confidence intervals (CIs) within a margin of error of 10% (Quatrics, Provo, UT).^21^

### Survey

We used the methods suggested by Burns et al.^22^ (best practices to survey healthcare providers), to develop and administer our online survey. Survey development began with a detailed review of the literature. We reviewed diabetes self-management and education consensus statements,^1^ and qualitative studies and surveys of the care experience of healthcare providers who support those with CKD.^12,23^ We also leveraged the results of patient-oriented research studies, including our own study on the care experience of patients with diabetes and advanced CKD in Ontario.^8^ Additionally, we reviewed the literature on special or nonconventional diabetes care programs that have been implemented and studied in this patient population.^17^

We next created a “table of specifications,” where we ensured that sufficient survey items/concepts had been generated from our literature search without duplication.^22^ This table was reviewed by all study investigators, as well as an external endocrinologist (JM) and two external diabetes educators (MD and JP) who worked in different Ontario DEPs. Based upon group feedback, KKC drafted preliminary survey questions. All investigators judged the appropriateness of each question included.

Then, the survey was pre-tested (for content) and pilot tested (for dynamics and flow) by diabetes educators (AM, MD, PB and JP). All team members reviewed the final draft for clinical sensibility (comprehensiveness).^22^

The survey was administered via Qualtrics software (www.qualtrics.com). Qualtrics uses Transport Layer Security (TLS) encryption for all transmitted data. Services are hosted by trusted data centers that are independently audited using the industry standard SSAE-16 method. Qualtrics servers are protected by firewall systems.^24^

Survey items are provided in Table 1 of the Supplemental Material.^22^ The first section of the survey included professional practice questions (city of practice, practice type, name of DEP, primary discipline, role as an education, and years in practice). The second section included multiple-choice questions focused upon the care experience of educators managing those with CKD and their associated challenges. The third section asked respondents about the availability of special programming for patients with diabetes and CKD in their communities. Respondents were also asked to identify some of the additional diabetes needs of this patient group. For three multiple-choice questions, respondents could select more than one response. For 10 questions, there was an option for free text or “other” entries (to capture other key concepts that we may not have considered).

**Table 1.** Characteristics of respondents (*n* = 122).

Characteristic	Number (%)
Practice setting	
Academic	6 (4.9)
Community	111 (91.0)
Missing	5 (4.1)
Primary discipline	
Dietitian	57 (46.7)
Nurse	58 (47.5)
Pharmacist	2 (1.6)
Other	5 (4.1)
Years in practice	
1–5 years	26 (21.3)
6–10 years	24 (19.7)
11–15 years	24 (19.7)
>15 years	48 (39.3)

### Ethics

Our study was approved by Western University’s Health Sciences Research Ethics Board. By submitting the survey, respondents implied consent to participate. Respondents could refuse to answer any question. Once the online surveys were completed, de-identified survey data were stored and analyzed on a password-protected hospital network drive, available only to study investigators.

### Analysis

We summarized results descriptively using proportions, and produced 95% CIs using Wilson’s score interval.^25,26^ For questions with free-text answers, we grouped responses into categories based upon their theme. If a free-text answer had a theme similar to pre-written multiple-choice answer, we grouped it with its relevant category. We used Microsoft Excel and SAS Version 9.3 (Cary, North Carolina) to organize the data and provide descriptive statistics.

## Results

### Participants

A flow diagram of participant recruitment is included in Figure 1 of the Supplemental Material. Between 21 May 2019 and 28 April 2020, there were 153 surveys initiated by diabetes educators across our province. In total, 122 unique, completed surveys were submitted. Although we could not calculate a total survey response rate (due to lack of knowledge of the total denominator of educators whom our survey reached), we were able to calculate a participation rate based upon the educators whom we sent our email directly to (participation rate∼79.2%).

### Characteristics of participants

Respondents worked across 61 different cities and 65 different DEPs. The majority worked in a community setting (111/122, 91%). Most were nurses (48%) and 39% had more than 15 years’ experience as a diabetes educator (Table 1). Most participants practiced in London Ontario (*n* = 15) followed by Toronto (*n* = 7), and Mississauga (*n* = 6) (Figure 1).

### Characteristics of non-responders

Although we were not able to capture the detailed characteristics of non-responders, there were fewer surveys submitted from the Northern and Eastern parts of our province (Figure 1).

### Educator experience with chronic kidney disease

Almost all (98%) respondents had some experience educating patients with CKD. Some treated more than 100 patients (45%). Most educators had not received special training (60%). Of the 40% who reported receiving special training, they had participated in in-service education (56%), attended courses (31%), conferences or seminars on diabetes management in CKD (7%).

Although educators had some experience with diabetes and CKD, only 5 (4.1%) felt “very confident” supporting these individuals. Most felt “somewhat confident” (66, 54.1%). Reasons for not feeling fully confident are presented in Table 2. Educators expressed particular uncertainly about managing patients on dialysis, and had reservations about providing patients with dietary advice.

**Table 2.** Reasons for not feeling “fully confident” managing diabetes in patients with chronic kidney disease.

Reasons	*n* respondents who reported	% Of all reasons reported	LCL	UCL
Lack confidence managing hemodialysis patients	71	31.4	25.7	37.7
Lack confidence managing peritoneal dialysis patients	68	30.1	24.5	36.4
Lack confidence providing dietary counseling	56	24.8	19.6	30.8
Lack confidence in drug dosing/side effects in CKD	42	18.6	14.1	24.2
Lack confidence making insulin adjustments	19	8.4	5.4	12.8
Not enough exposure or experience managing type 1 patients with CKD^a^	4	1.8	0.7	4.5
Not enough exposure to patients with CKD in general^a^	4	1.8	0.7	4.5
Lack confidence in hypoglycemia management/education^a^	2	0.9	0.2	3.2

Abbreviations: CKD, chronic kidney disease; LCL, lower confidence limit; UCL, upper confidence limit.

^a^Additional challenges reported in a free text “other” box.

Other challenges reported by participants included difficulty helping patients balance complex medical conditions, and facing socioeconomic barriers to treatment (Table 3). Participants also expressed difficulty scheduling appointments with patients and encouraging regular attendance. Most (85.2%) respondents hoped for more training/education to support those with diabetes and CKD.

**Table 3.** Challenges managing patients with chronic kidney disease.

Challenges	*n* respondents who reported challenge	% of all reported challenges	LCL	UCL
Balancing complex medical conditions	110	19.7	16.6	23.2
Socioeconomic barriers to treatment	100	17.9	15.0	21.3
Providing dietary advice to those who follow both kidney and diabetes diets	89	15.9	13.1	19.2
Difficulty scheduling/attending diabetes education sessions	66	11.8	9.4	14.8
Recurrent or severe hypoglycemia	50	9.0	6.9	11.6
Not having access to blood sugars at diabetes education appointments	47	8.4	6.4	11.0
Extreme hyperglycemia	45	8.1	6.1	10.6
Missed appointments	44	7.9	5.9	10.4
Difficulty collaborating with other providers who provide care for patients^a^	3	0.5	0.2	1.6
Emotional barriers^a^	1	0.2	0.0	1.0
Language barriers^a^	1	0.2	0.0	1.0
Difficulty accessing care/resources for patients^a^	1	0.2	0.0	1.0
Difficulty managing glycemic variability^a^	1	0.2	0.0	1.0

Abbreviations: LCL, lower confidence limit; UCL, upper confidence limit.

^a^Additional challenges reported in a free text “other” box.

### Availability of specialized diabetes support programs

Eighty-two percent of respondents were unaware of any specialized diabetes programming for those with CKD in their communities. Of those who noted the existence of special programs and services, the most commonly reported were outreach diabetes support programs (e.g., diabetes care provided in the dialysis unit). Some respondents commented upon the existence of interdisciplinary diabetes/kidney care clinics, and group/individual education tailored to those with diabetes and CKD (Supplemental Material Figure 1).

### Suggested patient needs

Almost all respondents (90%) felt that patients with diabetes and CKD needed more diabetes support than what was currently provided to them. Respondents felt patients would likely benefit from focused efforts to coordinate their care, more education, and self-management support (Table 4). Seventy-four percent of respondents reported that they would be interested in participating in new programming to support these individuals.

**Table 4.** Most important care need for those with diabetes and CKD (*n*=122).

Care need	*n*	%	LCL	UCL
Care coordination	42	34.4	26.6	43.2
More self-management support	20	16.4	10.9	24.0
More education about sick day management of diabetes medications	12	9.8	5.7	16.4
Resource navigation (e.g., access to foot care resources/personnel)	10	8.2	4.5	14.4
Hypoglycemia management	4	3.3	1.3	8.1
Support managing multiple comorbidities^a^	1	0.8	0.1	4.5
Medication support^a^	1	0.8	0.1	4.5

Abbreviations: CDE, certified diabetes educator; CKD, chronic kidney disease; LCL, lower confidence limit; UCL, upper confidence limit.

^a^Additional needs reported in free text “other” boxes.

## Discussion

### Main findings

Diabetes educators who responded to our survey had experience supporting patients with CKD but identified challenges in the provision of their care. Respondents noted that patients had complex medical issues; those with CKD and diabetes are in fact, at increased risk of hospitalization, morbidity and mortality.^27^ Both care providers and patients have expressed difficulty managing and prioritizing diabetes when multiple competing health issues are present.^13^ We also learned that respondents had difficulty providing patients with dietary advice as diabetes and kidney diets often conflict. Further, respondents noted socioeconomic barriers to treatment^7,28^; these barriers can limit access to diabetes technology and lower health literacy and self-management skills.^9^

In our survey, we also learned that educator respondents saw value in more programming to support this patient population. In busy outpatient clinics, providers may not have time to address the care needs of patients with multiple comorbidities, leading to care fragmentation.^13^ Improvements in diabetes care coordination (i.e., deliberate organization of patient care activities to facilitate the appropriate delivery of health care services)^29^ was frequently suggested as a need for those with CKD and diabetes.

### Relationship to other literature

There have been no published studies on the clinical care experience of diabetes educators supporting patients with CKD. However, our findings align with studies on the care experience of other healthcare professionals.^(12)^ Like educators, family doctors and specialists have noted gaps in their knowledge of diabetes/CKD management, and have expressed difficulty with dietary counseling.^(12,30,31)^ Dietary counseling may be especially difficult in those with CKD and diabetes as there is uncertainty about optimal dietary protein, animal and plant-based proteins, the role of complex carbohydrates, and the impact of special diets (e.g., the Mediterranean diet) on those with CKD.^30^ Moreover, dietary restrictions required by the kidney diet frequently contradict recommendations for the diabetes diet^12,31^; diets high in fiber and low in glycemic index are high in potassium and phosphorus, two key dietary restrictions in CKD.^31^

Like family doctors and specialists, we also learned that educators believe this patient population requires more care, with a particular emphasis on education,^12^ and care coordination. While we did not elicit the type of education that might be helpful to patients, in previous studies it has been suggested patients might be in need of more understanding of their illness, its treatments, complications, and the importance of remaining adherent to drugs, diet, and lifestyle.^3,12^ Education programs focused upon these concepts might improve patients’ self-management behavior, quality of life, glycemic control, and kidney function.^32–34^ The importance of coordinated care or care organized around patients with diabetes has been extensively highlighted in clinical practice guidelines.^35^ In previous studies, care providers have also echoed its importance. While care coordination is typically outside the role of the educator (involves organizing personnel and resources needed to carry out all required patient care activities),^29^ some suggest that primary care physicians are the ideal coordinator (with the support of specialists).^12^ Including allied health professionals like educators in “circles of care” might promote a stronger team-based approach. Previous studies have also suggested that interdisciplinary care clinics and outreach support programs which include allied health care providers might also promote care coordination.^17,23,32,36–38^

### Strengths and limitations

Our study was province-wide, and we used multiple pathways (DEPs, Section Chairs) to recruit educator participants. We created a comprehensive survey based upon detailed literature review and our own clinical and research experience with this patient group. We pre- and pilot tested our survey using recommended methods.^22^

In terms of limitations, we strongly relied upon publicly available email for recruitment, and we were not able to include DEPs with no email address. However, we did reach out to DEPs and Section Chairs where we could by telephone. Also, not all DEPs and Section Chairs acknowledged our recruitment email, and so we do not know the true denominator of diabetes educators reached. While we were able to estimate a participation rate based upon the known number of educators who individually received our email, this was an overestimate of our true response rate due to our sampling methods.

Survey studies are also subject to non-response bias^39^; educators who did not respond may have had different care challenges or no challenges at all. Also, our survey questions were categorical and responses could not be expanded upon. However, we did provide a number of opportunities for free-text answers which we summarized in our results.^22^

In addition, we could not fully account for participant education/certification in our analysis, though we did ask that the survey be completed by certified diabetes educators or CDEs, who are licensed care providers who must complete board testing and continuing education requirements every 5 years to maintain certification. Moreover, we did not ask whether nurse educators had nursing degrees, and participants may have had different educational backgrounds (e.g., university bachelor’s degree versus diploma) which may have influenced their responses. It is also possible that those who responded as dieticians may have been nutritionists. We did however ask that only respondents with at least 1 year in practice as a diabetes educator participate, and we ascertained their duration of practice, and the number of patients with CKD they had supported. It also must be emphasized that despite their background and education, results are still relevant as educator respondents were those currently and actively supporting patients with diabetes and CKD in our province.

The COVID-19 pandemic also negatively impacted our project. We had to lengthen the duration of our study due to on-site research personnel restrictions, and closed DEC offices. We fully expect that the pandemic also influenced response rates, particularly in 2020. Finally, results are most generalizable to educators from Ontario, Canada, particularly those from Southwestern Ontario. That being said, diabetes educators across other parts of our country are certified similarly, have similar scopes of practice, and work in similar clinical settings as in our region^20^

### Implications

Our survey of diabetes educator has implications for both clinical practice and research. From a clinical standpoint, it builds upon prior knowledge that care professionals need more support/education to manage patients with complex comorbidities. It newly identifies their need for more education about the intersection between diabetes and hemo- and peritoneal dialysis, balancing the diabetes and kidney diet, and hypoglycemia management in this population. Continuing professional development opportunities, practice papers and manuscripts, special training modules in diabetes and CKD might be of use.^16,30,40^

Our study may also be useful to researchers. While some communities already have access to special programming for patients CKD and diabetes, including dialysis-based outreach and interdisciplinary care clinics, participants confirmed that more programming is needed. Researchers might study the utility of unique programs to care for patients with CKD, particularly those that are co-designed with key stakeholders (i.e., diabetes educators and physicians), and formally study and evaluate them.^41^

## Conclusions

Our survey of diabetes educators adds to a growing body of literature suggesting the need to provide more support to both patients living with complex health conditions and their care providers.^7,8,12,23,27,42^ Our study suggests that educators might benefit from extra training and education about the interactions between kidney disease and diabetes, and that we might continue to make efforts to create and study new care programming that is relevant and needed by these patients and their providers.

## Supplemental Material

sj-pdf-1-cob-10.1177_26335565211062758 – Supplemental Material for Providing diabetes education to patients with chronic kidney disease: A survey of diabetes educators in Ontario, Canada Click here for additional data file. Supplemental Material, sj-pdf-1-cob-10.1177_26335565211062758 for Providing diabetes education to patients with chronic kidney disease: A survey of diabetes educators in Ontario, Canada by Kristin K Clemens, Alexandra M Ouédraogo, Selina L Liu, Paulina Bleah, Amanda Mikalachki and Tamara Spaic in Journal of Comorbidity
