# Supplementary material for: Providing diabetes education to patients with chronic kidney disease: A survey of diabetes educators in Ontario, Canada
Source: J Multimorb Comorb. 2021 Dec 13;11:26335565211062758. doi: 10.1177/26335565211062758 (PMC8671669; doi:10.1177/26335565211062758)
Supplement: sj-pdf-1-cob-10.1177_26335565211062758 – Supplemental Material for Providing diabetes education to patients with chronic kidney disease: A survey of diabetes educators in Ontario, Canada [file sj-pdf-1-cob-10.1177_26335565211062758.pdf]

Supplemental Material

Figure 1. Flow diagram of participant recruitment

Table 1. Survey items

Figure 2. Community programs to support diabetes and CKD

Figure 1. Flow diagram of participant recruitment

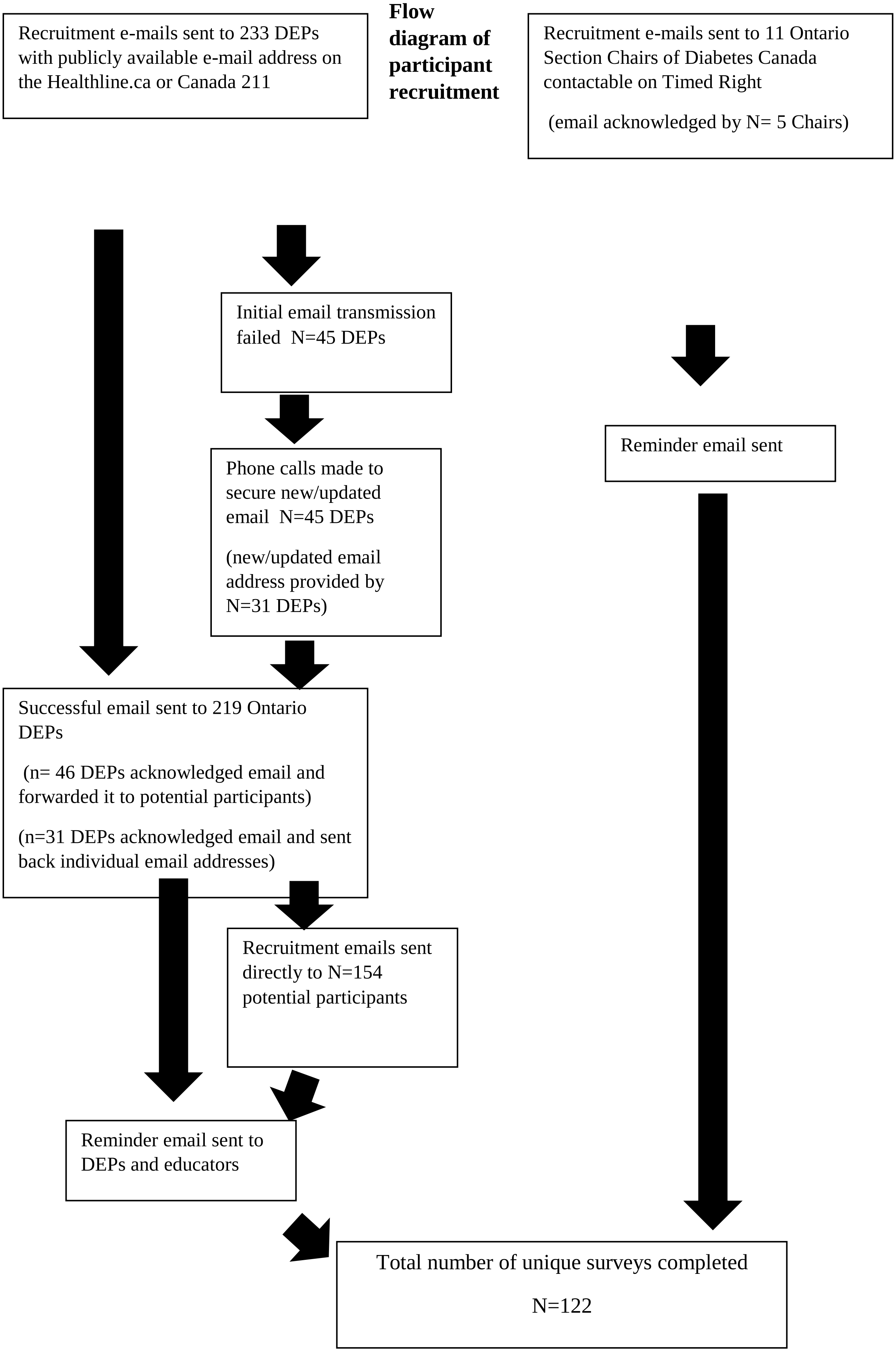

Table 1. Survey items

**Demographics**
City: ______________
Practice type: □Academic □Community
Diabetes Education Centre (please name): ____________________
Primary discipline: □Nurse □Pharmacist □Dietitian □Other ___________
Role: □Direct patient care □Administration □Research □Government □Other_________
Years in practice: □<1 year □1-5years □6-10 years □11-15 years □ >15 years
**SECTION A**
**1. Do you have *any* clinical experience managing patients with DM and CKD (i.e. eGFR <60 ml/min/1.73m^2)?**
□ Yes
□ No (skip to question 2)
**a. If yes, please estimate the number of patients with DM and CKD you have treated.**
□ <10
□ 10-50
□ 51-100
□ >100
**b. If yes, please indicate the type of patients you have managed (please check all that apply).**
□ DM1 and CKD, not using dialysis
□ DM1 using peritoneal dialysis
□ DM1 using hemodialysis
□ DM2 and CKD, not using dialysis
□ DM2 using peritoneal dialysis
□ DM2 using hemodialysis
□ Other (please specify) ___________________________________
**2. Have you had any formal training to help you manage patients with CKD and DM?**
□ Yes
□ No (skip to question 3)
**a. If yes, please describe the type training (please check all that apply)**
□ In-service training
□ Formal course
□ Other (please describe) _________________________
**3. What challenges do you face treating patients with CKD and DM? (please check all that apply)**
□ Providing dietary advice to those who follow both kidney and diabetes diets
□ Missed in-person appointments
□ Not having access to blood sugars at appointments
□ Extreme hyperglycemia
□ Recurrent (<2 times per week) or severe hypoglycemia
□ Balancing complex medical conditions
□ Socioeconomic barriers
□ Difficulty scheduling/attending diabetes education sessions due to frequent medical appointments

□ Other (please describe) _______________
□ Not applicable (i.e. I have *never* had to treat patients with CKD and DM)
**4. How confident do you feel managing patients with CKD and *DM2*?**
□ Very confident
□ Confident
□ Somewhat confident
□ Not confident
□ Uncertain
**5. How confident do you feel managing patients with CKD and *DM1?***
□ Very confident
□ Confident
□ Somewhat confident
□ Not confident
□ Uncertain
**6. If you are *not fully* confident managing patients with CKD and DM, please indicate why (check all that apply)?**
□ Lack confidence providing dietary counselling to this population
□ Lack confidence making insulin adjustments
□ Lack confidence in drug dosing/side effects
□ Lack confidence in hypoglycemia management/education
□ Lack confidence managing hemodialysis patients
□ Lack confidence managing peritoneal dialysis
□ Other (please describe) _______________
□ Not applicable (i.e. I am *fully confident* managing patients with CKD and DM)
**7. Do you feel that YOU need more support/training to manage patients with DM and CKD?**
□ Yes
□ No
□ Uncertain
**SECTION B**
**8. Are there specific diabetes support programs available to patients with CKD in your communities (e.g. dedicated CKD/DM education classes, diabetes outreach programs in CKD clinics/dialysis centres)?**
□ Yes
□ No (skip to question 9)
□ Uncertain
**a. If yes, please indicate the type of program(s)**
□ Group education for CKD/DM patients
□ Individual diabetes education for CKD/DM patients
□ CKD/DM outreach programs (e.g. provision of support outside of the diabetes education centre)
□ Other (please describe): _________________
**b. If yes, who carries out these programs?**
□ CDE/RD

□ CDE/RN
□ Nurse practitioner
□ Physician
□ Pharmacist
□ Other (please specify): ______________________
**9. Were there previously any diabetes support programs for CKD/DM patients in your community?**
□ Yes
□ No (skip to question 9)
□ Uncertain
**b. If yes, please indicate the type of program.**
□ Specific group education class for CKD/DM patients
□ Individual diabetes education/nutrition support for CKD/DM
□ CKD/DM outreach programs (e.g. provision of support outside of the diabetes education centre)
□ Other (please describe): ___________________________________
**c. Why were the programs discontinued?**
□ Patients didn't participate
□ No funding to continue
□ No personnel to continue the program
□ Unsure
□ Other (please specify) ________________________
**10. Do you see a need for more efforts to support patients with DM and CKD?**
□ Yes
□ No
□ Uncertain
If no, why? ____
**11. If yes, what do you feel would be most important to offer?**
□ Glycemic management
□ Management of hypoglycemia
□ Sick day management of diabetes medications
□ Self-management support
□ Care coordination
□ Resource navigation (e.g. access to foot care resources/personnel)
□ Foot screening
□ Screening reminders (e.g. vision)
□ Other (please describe) ____________________
**12. If there was funding to support you, would you be interested in helping with a diabetes support program for these individuals?**
□ Yes
□ No
□ Uncertain

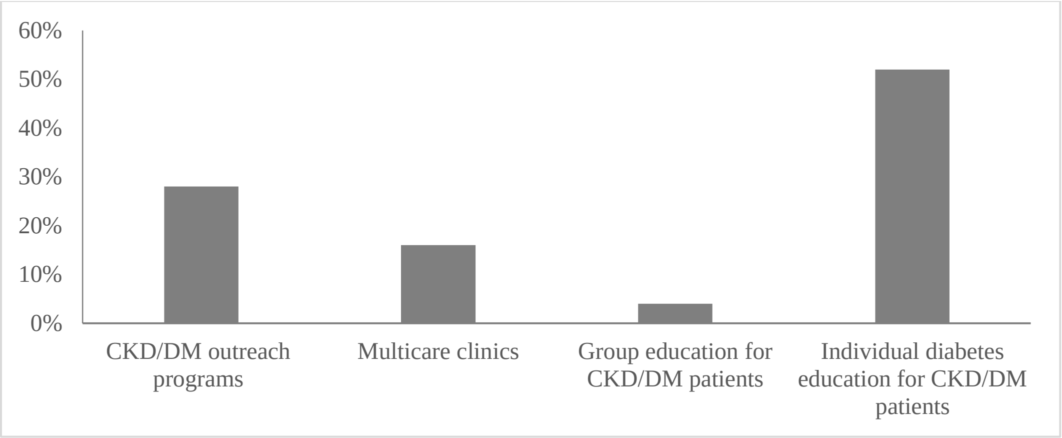

Figure 2. Community programs to support diabetes and CKD
